# Targeted TET oxidase activity through methyl‐CpG‐binding domain extensively suppresses cancer cell proliferation

**DOI:** 10.1002/cam4.830

**Published:** 2016-07-25

**Authors:** Yasuhiko Mizuguchi, Yuriko Saiki, Akira Horii, Shinichi Fukushige

**Affiliations:** ^1^ Department of Molecular PathologyTohoku University School of MedicineSendaiMiyagiJapan; ^2^ Center for Regulatory Epigenome and DiseasesTohoku University School of MedicineSendaiMiyagiJapan

**Keywords:** DNA demethylation, DNA methyltransferase inhibitor, hypermethylated genes, methyl‐CpG‐binding domain, ten‐ eleven translocation proteins

## Abstract

DNA methyltransferase (DNMT) inhibitors are epigenetic drugs used to treat myelodysplastic syndrome. They not only induce DNA demethylation but also have significant cytostatic and cytotoxic effects; however, the relationships between these characteristics have not been established yet due to the lack of a method to induce only DNA demethylation. Herein, we show that a fusion protein comprised of the methyl‐CpG‐binding domain (MBD) and the catalytic domain of Ten‐eleven translocation protein 1 (TET1‐CD) globally demethylates and upregulates a number of methylated genes. These upregulated genes frequently contained CpG islands (CGIs) within ± 1000 bp of the transcription start site (TSS). Interestingly, 65% of the genes upregulated fivefold or more by MBD‐TET1‐CDwt were also reactivated after treatment with a DNMT inhibitor, 5‐azacytidine (Aza‐CR), suggesting that gene reactivation by both methods primarily shares the same mechanism, DNA demethylation. In order to examine whether DNA demethylation affects the growth of cancer cells, we have established a tetracycline inducible system that can regulate the expression of MBD‐TET1‐CDwt in a prostate cancer cell line, LNCaP. The induction of MBD‐TET1‐CDwt demethylated and upregulated glutathione S‐transferase pi 1 (*GSTP1*), one of the hypermethylated genes in prostate cancer. In accordance with the reactivation of methylated genes, induction of MBD‐TET1‐CDwt extensively suppressed the growth of LNCaP cells through G1/S arrest. These results clearly indicate that TET oxidase activity recruited at methyl‐CpG sites through MBD induces reactivation of hypermethylated genes by DNA demethylation and allows us to analyze the effect of only global DNA demethylation in a wide variety of cancer cells.

## Introduction

Two DNA methyltransferase (DNMT) inhibitors, 5‐aza‐2'‐deoxycytidine (decitabine) and its analog 5‐azacytidine (azacitidine), are currently used as epigenetic therapeutic drugs for patients with a subset of cancers such as myelodysplastic syndromes (MDS) and acute myelogenous leukemias (AML) 1, 2. When one of these base analogs is incorporated into DNA, DNMT becomes covalently linked to the modified base; 3 this triggers the proteasomal degradation of DNMTs, 4 leading to global demethylation and gene reactivation. These DNMT inhibitors induce significant cytostatic and cytotoxic effects. These characteristics have also been linked to the formation of covalent adducts between incorporated azacytosine bases and DNMTs, which cause stalled replication forks and pronounced changes in the cell cycle 5. Therefore, it remains elusive to what extent DNA demethylation contributes to these characteristics. In order to assess the effects of global DNA demethylation, one of the most commonly used approaches is DNMT knockout studies. However, because DNMTs have the potential to repress transcription independently of their methylating activities 6, 7, this approach may not suffice. Therefore, we sought to use the catalytic activity of the Ten‐eleven translocation (TET) family proteins in order to demethylate and upregulate various methylated genes in the genome.

TET proteins are Fe(II)‐ and *α*‐ketoglutarate (*α*‐KG)‐dependent dioxygenases that successively convert 5‐methylcytosine (5‐mC) into 5‐hydroxymethylcytosine (5‐hmC), 5‐formylcytosine (5‐fC), and 5‐carboxylcytosine (5‐caC). These oxidized methylcytosines are believed to be intermediates in the process of 5‐mC demethylation 8. Previous studies have reported that overexpression of the TET1 catalytic domain (TET1‐CD) in human embryonic kidney cell line 293T (HEK293T) can induce DNA demethylation and gene reactivation in exogenous nonreplicable DNA reporters and even in endogenous genomic loci with limited levels 9, 10, 11. Interestingly, overexpression of full length TET1 (TET1‐FL) did not induce global DNA demethylation in HEK293T cells because of the preferential binding of TET1‐FL to unmethylated CpG islands (CGIs) through its CXXC domain 11. These results indicate that a system targeting TET1‐CD at hypermethylated promoter regions is necessary to demethylate and upregulate a number of methylated genes. We previously used the methyl‐CpG‐binding domain (MBD) 12 and developed a novel method that can reactivate silenced genes by promoter hypermethylation; this method is called “methyl‐CpG targeted transcriptional activation,” MeTA for short 13, 14, 15, 16. Reactivation is achieved by the introduction of a plasmid which carries a fusion gene comprised of MBD and the transcriptional activation domain (TAD). In this study, we constructed a fusion gene between TET1‐CD and MBD to ask (1) whether this fusion gene can demethylate and upregulate a wide variety of methylated genes, and (2) whether it can suppress the growth of prostate cancer cells.

## Materials and Methods

### Strain and plasmids

Four DNA fragments were used for construction of the five plasmids indicated in Figure S1a; 3xFLAG from p3xFLAG‐CMV‐10 (Sigma, St. Louis, MO, USA), MBD (codons 144 through 230) from pcDNA‐MBD2 13, TET1‐CDwt (codons 1418 through 2136) from pcDNA3b‐hTET1‐CD‐HA 10, and TET1‐ CDmut (codons 1418 through 2136, D1674A) from pcDNA3b‐hTET1‐CDmut‐HA 10. The latter two were kindly provided by Drs. J.U. Guo and H. Song (Johns Hopkins University School of Medicine). *E. coli* strain DH5*α*F' was used for plasmid preparation. Relevant fragments were PCR‐amplified using KOD DNA polymerase (Toyobo, Osaka, Japan) and were cloned into the pcDNA6/Myc‐His A vector (Invitrogen, Carlsbad, CA, USA). Nucleotide sequences of the plasmids were confirmed. Nucleotide sequences of the PCR primers are available upon request to the authors.

### Cell culture, plasmid transfection, and establishment of stable cell lines

HEK293T was grown in Dulbecco's modified Eagle's medium (Wako, Osaka, Japan) supplemented with 10% fetal bovine serum (FBS) (Invitrogen) as described previously 17. A human prostate cancer cell line, LNCaP, was purchased from American Type Culture Collection (ATCC, Manassas, VA, USA) and was grown in RPMI‐1640 medium (Sigma) supplemented with 10% FBS. HEK293T cells were seeded in 6‐well dishes, and transfection was performed with an aliquot of 4 *μ*g of each plasmid using Lipofectamine 2000 Transfection Reagent (Invitrogen) according to the manufacturer's instructions. To obtain stable transfectants, cells were incubated in medium supplemented with 10 *μ*g/mL blasticidin S (Invitrogen) after 48 h of transfection. Individual colonies were picked and subsequently expanded in 6‐well dishes. LNCaP cells that allow tetracycline‐regulated MBD‐TET1‐CDwt or MBD‐TET1‐CDmut expression were constructed in two steps using the T‐REx system (Invitrogen). First, LNCaP cells were seeded in 6‐well dishes and transfected with 1 *μ*g of pcDNA6/TR plasmid, using Lipofectamine Reagent. Two cell lines (LNCaP_TR9 and LNCaP_TR15) were selected with RPMI‐1640 medium containing 7.5 *μ*g/mL blasticidin S because transient expression of pcDNA4/TO/myc‐His/lacZ (Invitrogen) resulted in the extensive induction of *β*‐galactosidase activities upon addition of tetracycline (85‐fold and 138‐fold, respectively). Second, a pcDNA4/TO/3xFLAG‐MBD‐TET1‐CDwt or pcDNA4/TO/3xFLAG‐MBD‐TET1‐CDmut plasmid was digested with *Pvu*I and transfected into these selected cells to give four cell lines; LNCaP_TR9‐MBD‐TET1‐CDwt #17, LNCaP_TR9‐MBD‐TET1‐CDmut #9, LNCaP_TR15‐MBD‐TET1‐CDwt #16, and LNCaP_TR15‐MBD‐TET1‐CDmut #24. These cell lines were maintained in RPMI‐1640 medium containing 7.5 *μ*g/mL blasticidin and 500 *μ*g/mL zeocin.

### Immunofluorescence staining

Cells were washed, fixed, and incubated with each monoclonal antibody at 48 h after plasmid transfection as described previously 18. Dilution of primary antibodies was 1:1000 for anti‐FLAG M2 (Sigma) and 1:500 for anti‐5hmC (ActiveMotif, Carlsbad, CA, USA). Indirect detection of primary antibodies was achieved by 60 min‐incubation with 1:100 diluted secondary antibodies: FITC‐labeled goat anti‐mouse IgG (Zymed 62‐6511, Invitrogen, Carlsbad, CA, USA) and Rhodamine‐labeled goat anti‐rabbit IgG (Chemicon AP‐187R, EMD Millipore, Darmstadt, Germany). Cells were stained for 10 min with 0.5 *μ*g/mL 4', 6'‐diamidino‐2‐phenylindole (DAPI). Images were acquired on an Olympus BX60 fluorescence microscope fitted with a charge‐coupled device camera (Photometrics, Tucson, AZ, USA) and controlled by a Macintosh computer running the Quips mFISH software (Vysis Inc., Downers Grove, IL, USA).

### Western blotting

Western blotting was performed according to the previous protocols 19. Membranes were probed, using mouse monoclonal anti‐FLAG M2 antibody at 1:1000 dilution and mouse monoclonal anti‐*β*‐actin (Sigma) antibody at 1:3000 dilution. Detection was carried out using peroxidase‐conjugated anti‐mouse IgG (GE Healthcare, Buckinghamshire, UK) antibody. Signals were visualized by reaction with ECL Detection Reagent (GE Healthcare) and digitally processed using LAS 4000 (Fujifilm, Tokyo, Japan).

### Reverse transcription polymerase chain reaction (RT‐PCR)

Each aliquot of 2 *μ*g of total RNA was reverse transcribed, and the single stranded cDNA was synthesized using a High Capacity cDNA Reverse Transcription Kit (Applied Biosystems, Foster City, CA, USA). RT‐PCR amplifications using intron‐spanning primers were performed as described 20, and their products were run in 4% agarose gels. The *B2M* was used as the internal control 21. Analyzed were the following genes: *SOX17*,* MLH1*,* MAL*,* MT1M*, and *TRH*. Nucleotide sequences of the primers for *SOX17* are as follows: *SOX17*‐F1, 5'‐CGAGTTGAGCAAGATGCTGG‐3'; *SOX17*‐R1, 5'‐GTCCTGCATGTGCTGCACG‐3'. Other primers are previously described 13, 15.

### Sodium bisulfite sequencing

Bisulfite treatment of the relevant genomic DNA was carried out with EpiTect Bisulfite Kit (Qiagen, Valencia, CA, USA). PCR was performed to amplify the sequences around the promoter regions of *TRH* and *MAL*. PCR products were subcloned into pKRX vector by TA cloning 22 and sequenced on an ABI sequencer with dye terminators (Applied Biosystems). Primers used for bisulfite sequencing are described previously 15 and in Table S2.

### Quantitation of 5‐mC and 5‐hmC

EpiMark 5‐hmC and 5‐mC Analysis Kit (New England Biolabs, Ipswich, MA, USA) was used to quantitate the amounts of C, 5‐mC, and 5‐hmC in the *MAL* promoter region. Genomic DNA was treated with T4 *β*‐glucosyltransferase (T4‐BGT) and digested with restriction endonuclease *Hpa*II or *Msp*I. These endonucleases recognize the same CCGG sequence, but the former cannot cut the DNA containing a methylated CpG sequence, whereas the latter can cut irrespective of methylation status. Then quantitative genomic PCR using SYBR Green was performed for quantifying the methylation status of the CpG site in CCGG sequences. Nucleotide sequences of this PCR are as follows: *MAL*8‐s1‐F, 5'‐TTGAGTTTGTGAGTGGCTCCT‐3'; *MAL*8‐s1‐R, 5'‐GGACTTCCCTGGCTTAGTC‐3'.

### Microarray

Microarray analyses were performed according to methods described previously 15. Cy‐3 labeled cRNAs from HEK293T stable cell lines were hybridized to Agilent Human Gene Expression 4x44K v2 Microarray Kit. The cut‐off value for fivefold upregulation was employed for selection of the genes. High‐throughput microarray data are available in the Gene Expression Omnibus database (http://www.ncbi.nlom.nih.gov/geo) under the accession number: GSE74744.

### CGI analysis

We performed two types of searches; (1) between 2‐kb upstream from the transcription start site (TSS) to the end of the gene, and (2) between 1‐kb upstream from the TSS to 1‐kb downstream. The former was termed as CGI, and the latter as CGI ± 1000‐bp. The CpG islands searcher (http://cpgislands.usc.edu/) was used for Gardiner‐Garden and Frommer's criteria, %GC > 50, length > 200‐bp, and observed CpG/expected CpG > 0.6 23 and for Takai and Jones' criteria, %GC ≥ 55, length ≥ 500‐bp, and observed CpG/expected CpG ≥ 0.65 24.

### Cell proliferation assay

LNCaP‐derived 3 × 10^3^ cells were seeded in 24‐well dishes at day ‐1. Cells were treated with or without 1 *μ*g/mL tetracycline, and cell viability was assessed at days 0, 2, 4, 6, and 8 by alamarBlue assay (Invitrogen) as described previously 18. Experiments were performed in quadruplicate and repeated three times.

### Cell cycle analysis

Cells were grown in the presence of 1 *μ*g/mL tetracycline (replaced every other day) and were harvested at days 2, 4, 6, and 8. The cell cycle distribution of samples was determined by flow cytometric analysis (FACSCanto II; BD Biosciences) as described previously 25.

## Results

### The TET1‐CD fused to MBD extensively upregulates epigenetically silenced genes

**Figure 1:**
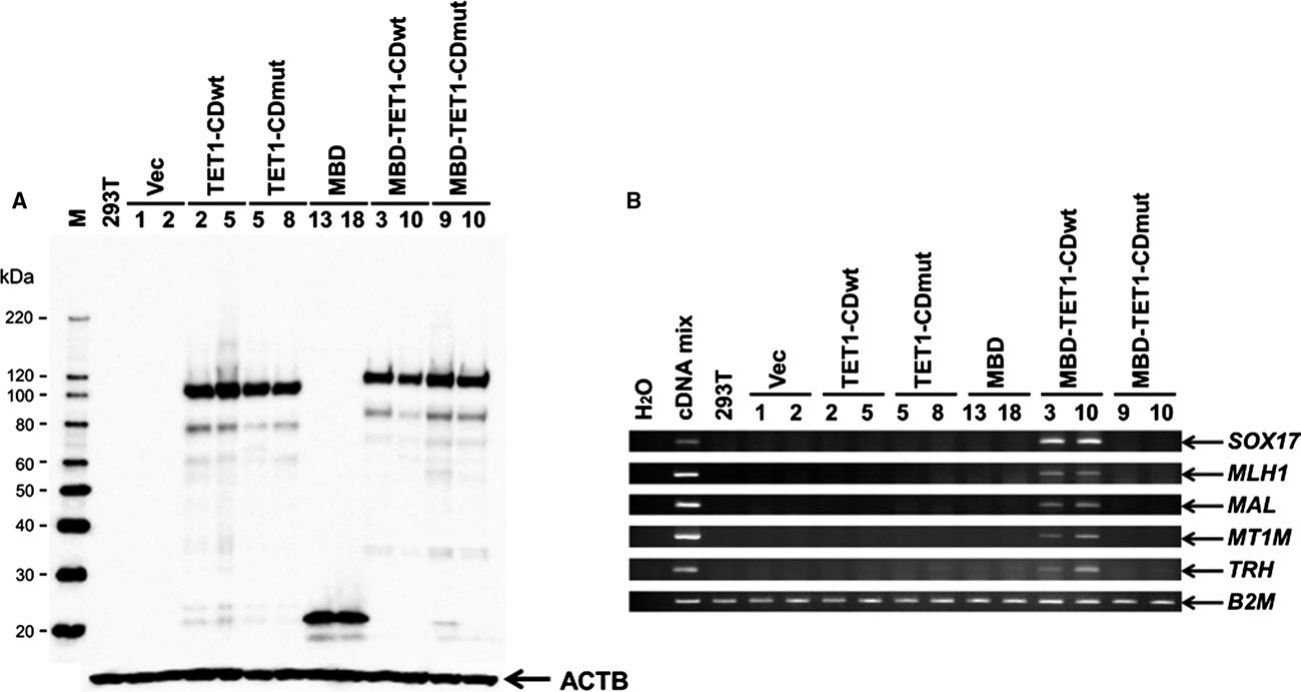
MBD‐TET1‐CDwt reactivates hypermethylated genes in HEK293T cells. (A) Western blotting analysis using anti‐FLAG antibody indicates the expression of each TET1‐CD fusion construct (Fig. S1A) obtained from two independently isolated HEK293T stable cell lines. ACTB is an endogenous control. (B) RT‐PCR analysis shows that only stable HEK293T cell lines containing MBD‐TET1‐CDwt #3 and #10 reactivates all five hypermethylated genes. *B2M* is an endogenous control. Lane “cDNA mix” is a positive control and uses a mixture of cDNAs from fetal brain, fetal liver, kidney, pancreas, lung, liver, ovary, fibroblast, and colon as the PCR template.

In previous studies, overexpression of TET1‐CD in HEK293T induced DNA demethylation and gene reactivation with limited induction levels 11. In order to maximize the effect of TET1‐CD, we invented a system that should recruit TET1‐CD at hypermethylated promoters in a positive manner by using MBD. We first constructed fusion genes that contain the wild‐type (wt) TET1 catalytic domain (TET1‐CDwt) as well as the catalytically inactive TET1‐CD mutant (TET1‐CDmut) with or without MBD and transiently transfected each DNA construct into HEK293T cells. Immunofluorescence analyses using anti‐5‐hmC antibody clearly indicated that TET1‐CDwt, but not TET1‐CDmut, is functional and produces 5‐hmC in cell nuclei (Fig. S1). Then we asked whether these fusion constructs could upregulate a number of methylated genes. Because insufficient transfection efficiency will mask the precise effects of TET1‐CD, we made two independent stable cell lines expressing each fusion construct, as shown in Figure 1A, and analyzed the expression of five methylated genes, including SRY (sex determining region Y)‐box 17 (*SOX17*)*,* mutL homolog 1 (*MLH1*)*,* myelin and lymphocyte protein (*MAL*)*,* metallothionein 1M (*MT1M*), and thyrotropin‐releasing hormone (*TRH*) 15. These five genes carrying CGIs at their promoter regions were randomly selected from our previous studies on hypermethylated genes that were confirmed by genomic bisulfite sequencing in HEK293T cells 13, 15. It is notable that only stable cell lines expressing MBD‐TET1‐CDwt extensively reactivated all five methylated genes; other constructs did not upregulate any methylated genes (Fig. 1B). These results suggest that the accumulation of TET1 catalytic activity at hypermethylated promoters through MBD facilitates the extensive upregulation of endogenous methylated genes.

### MBD‐TET1‐CDwt demethylates hypermethylated promoters

**Figure 2:**
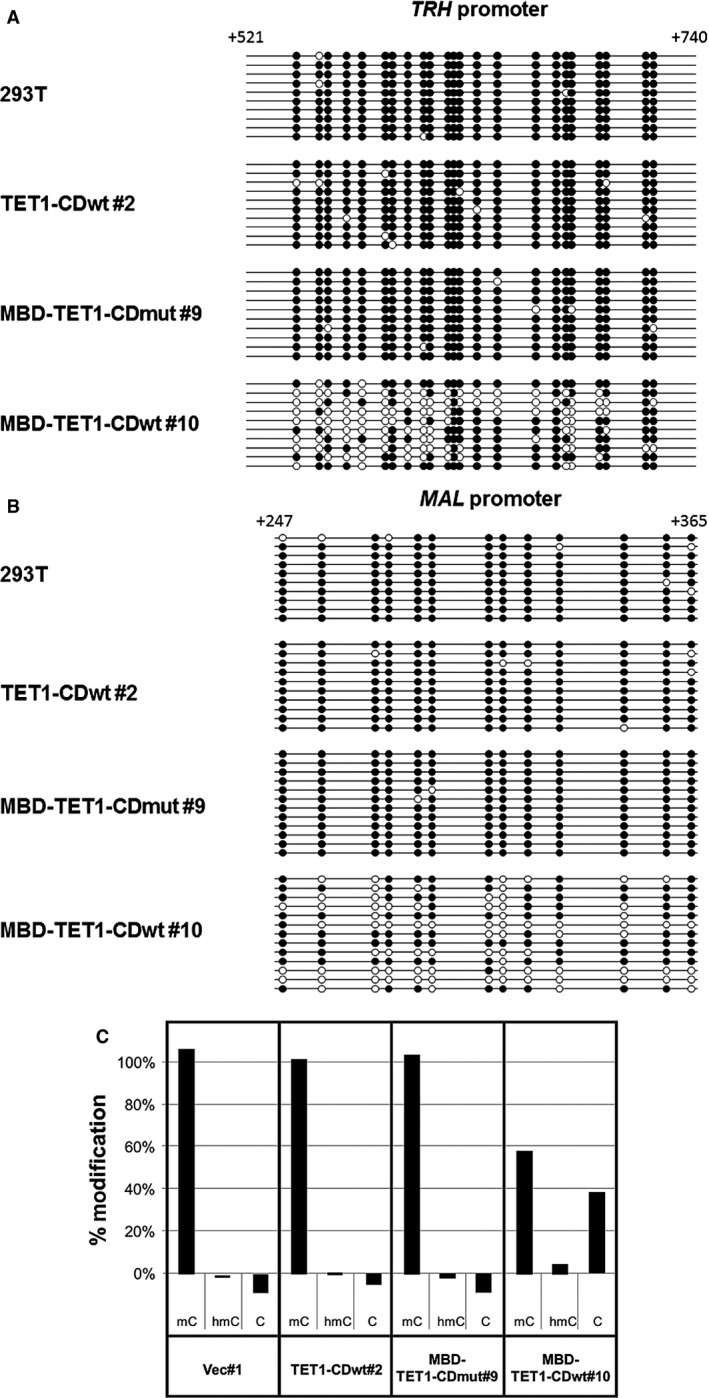
DNA demethylation occurs at hypermethylated promoters in cell lines expressing MBD‐TET1‐CDwt. Bisulfite genomic sequencing of *TRH* (A) and *MAL* (B) promoter regions is shown in parental cell line HEK293T, along with its derivatives, TET1‐CDwt #2, MBD‐TET1‐CDmut #9, and MBD‐TET1‐CDwt #10. Analyzed regions are located within the first intron of *TRH* and *MAL* genes. Note that both promoters are demethylated only in the MBD‐TET1‐CDwt cell line. Closed and open circles indicate the methylated and unmethylated CpG sites, respectively. (C) The differences in methylation status within a specific locus of the *MAL* promoter were analyzed and quantitated using EpiMark 5‐hmC and 5‐mC Analysis Kit. Note that C and 5‐hmC were seen only in the MBD‐TET1‐CDwt cell line.

We hypothesized that MBD‐TET1‐CDwt should upregulate a number of genes by demethylating their hypermethylated promoters. TET1 catalytic activity likely oxidizes 5‐mC with the help of MBD; subsequently, several mechanisms including passive DNA demethylation through replication 26 and active DNA demethylation through base excision repair (BER) 27 converts these oxidized methylcytosines into cytosines. In order to confirm DNA demethylation, we analyzed CpG sites in promoters of *TRH* and *MAL* by genomic bisulfite sequencing. Results are schematically shown in Figure 2. MBD‐TET1‐CDwt‐expressing HEK293T showed frequent demethylation, but the other two vector‐transfected stable cell lines along while parental HEK293T remained highly methylated. To further analyze the cause(s) of demethylation, we stoichiometrically examined 5‐mC and 5‐hmC in the *MAL* promoter, using the EpiMark 5‐hmC and 5‐mC Analysis Kit (Fig. 2C). When 5‐hmC occurs in the context of CCGG, this modification converts a cleavable *Msp*I site to a noncleavable one. Real‐time PCR analysis clearly indicates that production of 5‐hmC with extensive amounts of cytosine was observed only in the *MAL* promoter of MBD‐TET1‐CDwt #10.

In addition to hypermethylated promoters, distal, and proximal enhancer regions are critical to regulate gene expression and can be targeted by MBD‐TET1‐CDwt. Long interspersed nucleotide elements (LINEs) were also analyzed as an indicator for global DNA demethylation and we found that DNA demethylation only increased in MBD‐TET1‐CDwt #10 (data not shown).

### MBD‐TET1‐CDwt upregulates a number of genes with CGIs in the promoter

To understand the characteristics of genes upregulated by MBD‐TET1‐CDwt, we performed gene expression microarray analysis using two distinct, independently isolated transfectants obtained from each vector as follows: empty vector, TET1‐CDwt, MBD‐TET1‐CDwt, and MBD‐TET1‐CDmut. We first validated the adequacy of this system by the examining genes analyzed in Figure 1B. Signal intensities of all the analyzed genes (*SOX17*,* MLH1*,* MAL*,* MT1M* and *TRH*) were significantly higher in transfectants with MBD‐TET1‐CDwt #3 and #10 than in ones with the empty vector, TET1‐CDwt, or MBD‐TET1‐CDmut. Analysis using normalized intensity values clearly indicated that more genes are upregulated in cells containing MBD‐TET1‐CDwt in comparison with ones containing empty vector, TET1‐CDwt, or MBD‐TET1‐CDmut (Fig. S2). The number of genes upregulated by fivefold or more in transfectants with TET1‐CDwt, MBD‐TET1‐CDwt, and MBD‐TET1‐CDmut, when compared with empty vector, were 1, 60, and 6, respectively (Table 1 and S1). We searched the CGIs of each upregulated gene according to two accepted criteria: one by Gardiner‐Garden and Frommer 23 and the other by Takai and Jones 24. Interestingly, the frequency of CGIs within ± 1000‐bp of the TSS were significantly higher in genes upregulated by MBD‐TET1‐CDwt by the former criteria. Because the core region of about 1000‐bp centered at the TSS is generally unmethylated and is associated with open chromatin structure and transcriptional activation, these data may indicate that MBD‐TET1‐CDwt efficiently affects these distinct regions.

**Table 1:** Frequency of CpG islands in genes upregulated by three TET1‐CD constructs

	Takai and Jones		Gardiner‐Garden and Frommer		Total
	CGI (%)	CGI‐TSS ± 1000‐bp (%)	CGI (%)	CGI‐TSS ± 1000‐bp (%)	
TET1‐CDwt	1 (100)	1 (100)	1 (100)	1 (100)	1
MBD‐TET1‐CDwt	45 (75)	41 (68.3)	59 (98.3)	54 (90)	60
MBD‐TET1‐CDmut	4 (66.7)	3 (50)	5 (83.3)	4 (66.7)	6

CGI, CpG island; TSS, transcription start site.

### Genes upregulated by MBD‐TET1‐CDwt are frequently overlapped with ones upregulated by Aza‐CR

**Figure 3:**
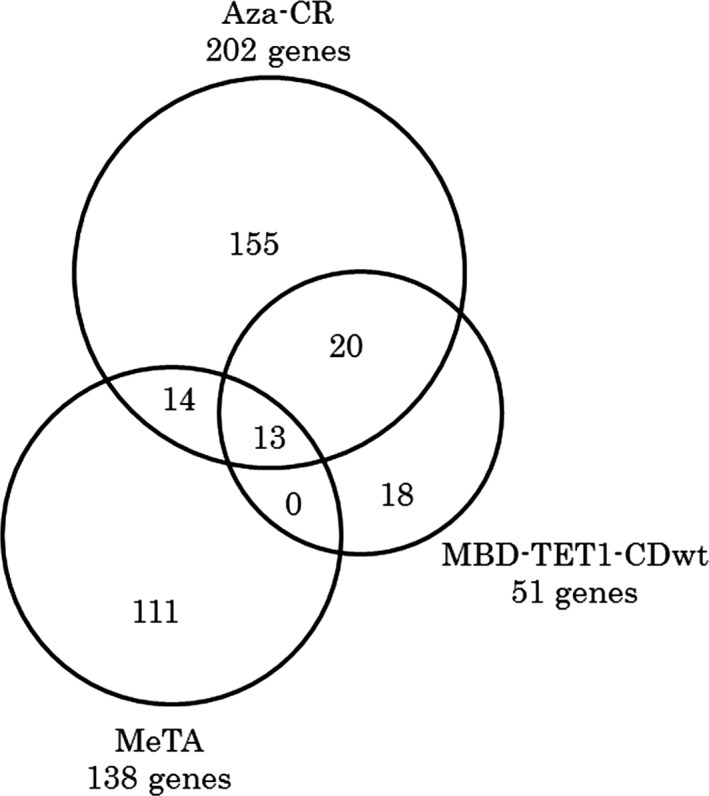
The genes upregulated by MBD‐TET1‐CDwt overlap with ones upregulated by Aza‐CR with a high frequency. The number of genes upregulated by fivefold or more in MBD‐TET1‐CDwt was 51. A Venn diagram shows that 33 of these 51 (65%) genes overlapped with ones upregulated by Aza‐CR.

We previously demonstrated that MeTA can identify different hypermethylated genes from those recognized by Aza‐CR and discussed the differences in reactivation mechanisms of these two methods. In the present study, we analyzed upregulated genes by three methods; Aza‐CR, MeTA, and MBD‐TET1‐CDwt. Because we used the Whole Human Genome DNA Microarray 4x44K v1 in a previous study and v2 in this study, we excluded the results of newly added probes in v2 to fairly compare the data. The genes upregulated fivefold or more were selected as shown by a Venn diagram (Fig. 3). Thirteen commonly upregulated genes were identified. It is notable that 33 of 51 (65%) upregulated genes by MBD‐TET1‐CDwt were also upregulated by Aza‐CR. This result may be caused by DNA demethylation as the common reactivation mechanism. To confirm this hypothesis, we analyzed methylation statuses of four cancer/testis (CT) antigen genes, *MAGEA2B*,* MAGEA8*,* MAGEA9*, and *XAGE1B* that were commonly upregulated by both MBD‐TET1‐CDwt and Aza‐CR. The genomic bisulfite sequencing analyses clearly demonstrated that the promoter regions of all four CT antigen genes were hypermetylated in HEK293T cells but were demethylated in MBD‐TET1‐CDwt‐expressing HEK293T cells. The representative results were shown in Figure S3. On the other hand, no genes upregulated by both MBD‐TET1‐CDwt and MeTA excluding Aza‐CR were found.

### Induction of MBD‐TET1‐CDwt demethylates and upregulates *GSTP1* gene in LNCaP cells

**Figure 4:**
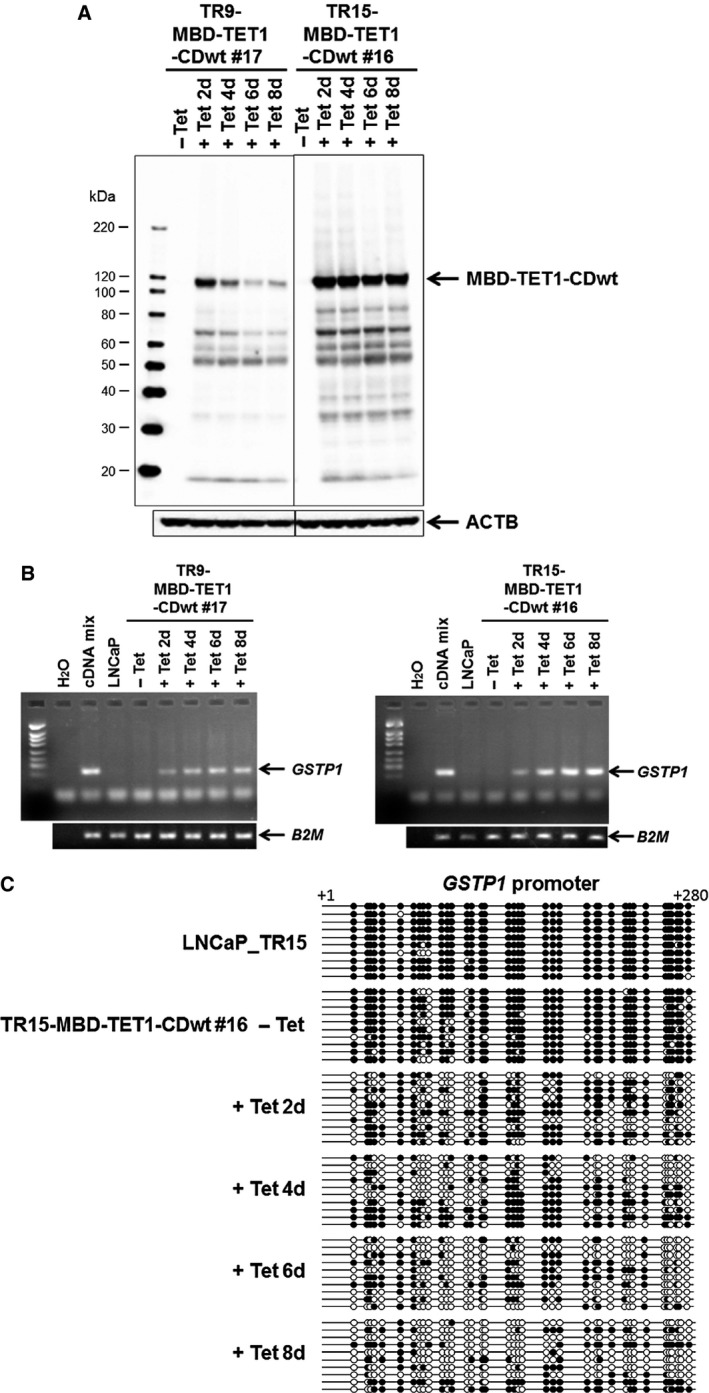
Inducible expression of MBD‐TET1‐CDwt reactivates the hypermethylated *GSTP1* gene through DNA demethylation. Immunoblotting analysis of MBD‐TET1‐CDwt protein using anti‐FLAG antibody (A). MBD‐TET1‐CDwt protein was induced after tetracycline (Tet) treatment in TR15‐MBD‐TET1‐CDwt #16 or TR9‐MBD‐TET1‐CDwt #17 cells. ACTB was used as an endogenous control. RT‐PCR analyses (B) indicate that the epigenetically silenced *GSTP1* gene was reactivated in response to MBD‐TET1‐CDwt induction by Tet addition. *B2M* was used as an endogenous control. (C) Bisulfite genomic sequencing of 37 CpG sites at the *GSTP1* promoter region is shown in the parental cell line, LNCaP_TR15, along with its derivative, TR15‐ MBD‐TET1‐CDwt #16 with or without Tet. Note that the *GSTP1* promoter was demethylated in TR15‐MBD‐TET1‐CDwt #16 cells in the presence of Tet. Closed and open circles indicate the methylated and unmethylated CpG sites, respectively.

To clarify whether DNA demethylation affects the growth of cancer cells, we established a tetracycline inducible system of MBD‐TET1‐CDwt in a human prostate cancer cell line, LNCaP. As shown in Figure 4A, MBD‐TET1‐CDwt inducible cell line LNCaP_TR15‐MBD‐TET1‐CDwt #16 extensively induced MBD‐TET1‐CDwt protein at 2 days after tetracycline addition compared to another cell line, LNCaP_TR9‐MBD‐TET1‐CDwt #17. Accordingly, glutathione S‐transferase pi 1 (*GSTP1*) that is known to be one of the frequently hypermethylated genes in prostate cancer, gradually upregulated its expression from day 2 after tetracycline addition (Fig. 4B). We further performed genomic bisulfite sequencing of the *GSTP1* promoter region that covers 37 CpG sites in exon 1 and intron 1 and identified that most of the CpG sites were methylated in the parental cell line LNCaP_TR15 and its derivative LNCaP_TR15‐MBD‐TET1‐CDwt #16 without tetracycline, whereas LNCaP_TR15‐MBD‐TET1‐CDwt #16 cells with tetracycline treatment showed prominent demethylation (Fig. 4C).

### Induction of MBD‐TET1‐CDwt suppresses cell growth in LNCaP

**Figure 5:**
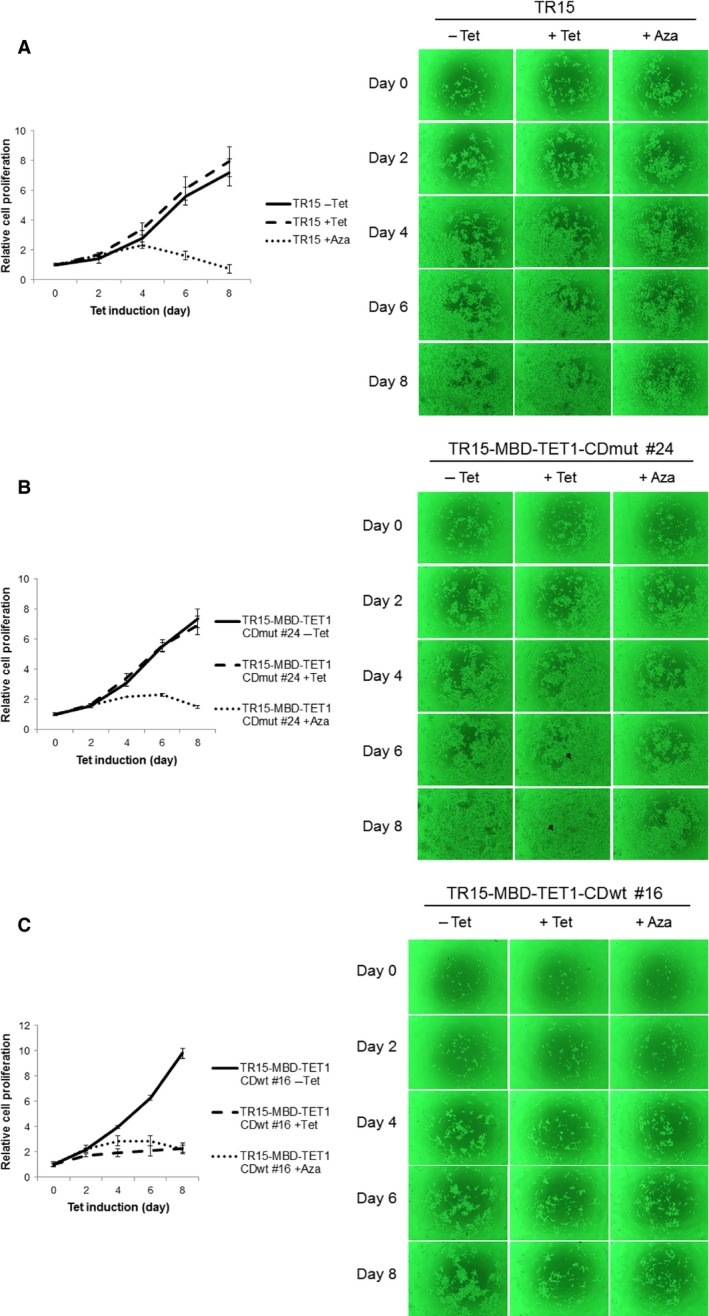
Inducible expression of MBD‐TET1‐CDwt suppresses the growth of LNCaP cells. Cell proliferation assay for LNCaP‐derived TR15 (A), TR15‐MBD‐TET1‐CDmut #24 (B), and TR15‐MBD‐TET1‐CDwt #16 (C) cells. Growth curves of TR15 (A) and TR15‐MBD‐TET1‐CDmut #24 (B) cells did not change either with or without tetracycline (Tet) addition. In contrast, Tet addition strongly inhibited cell proliferation of TR15‐MBD‐TET1‐CDwt #16 (C) cells. In the presense of Tet, MBD‐TET1‐CDwt protein is induced in TR15‐MBD‐TET1‐CDwt #16 (C) cells, whereas MBD‐TET1‐CDmut protein is induced in TR15‐MBD‐TET1‐CDmut #24 (B). The diagram in the right shows the light microscopic appearance (40× magnification). Cells were also treated with 1 *μ*mol/L 5‐aza‐2'‐deoxycitidine (Aza) and the Aza treatment induced the growth inhibition in all LNCaP‐derived cells.

We then analyzed the effects of MBD‐TET1‐CDwt induction on cell growth. We performed alamarBlue assays using the parental cell line (LNCaP_TR15), the MBD‐TET1‐CDmut inducible cell line (LNCaP_TR15‐MBD‐TET1‐CDmut #24), and the MBD‐TET1‐CDwt inducible cell line (LNCaP_TR15‐MBD‐TET1‐CDwt #16) with or without 1 *μ*g/mL tetracycline. Results are shown in Figure 5; only LNCaP_TR15‐MBD‐TET1‐CDwt #16 showed significant growth suppression between days 2–8 after tetracycline addition. We also used another set of cell lines and found that these results were reproducible, although the level of growth suppression was variable (Fig. S4).

**Figure 6:**
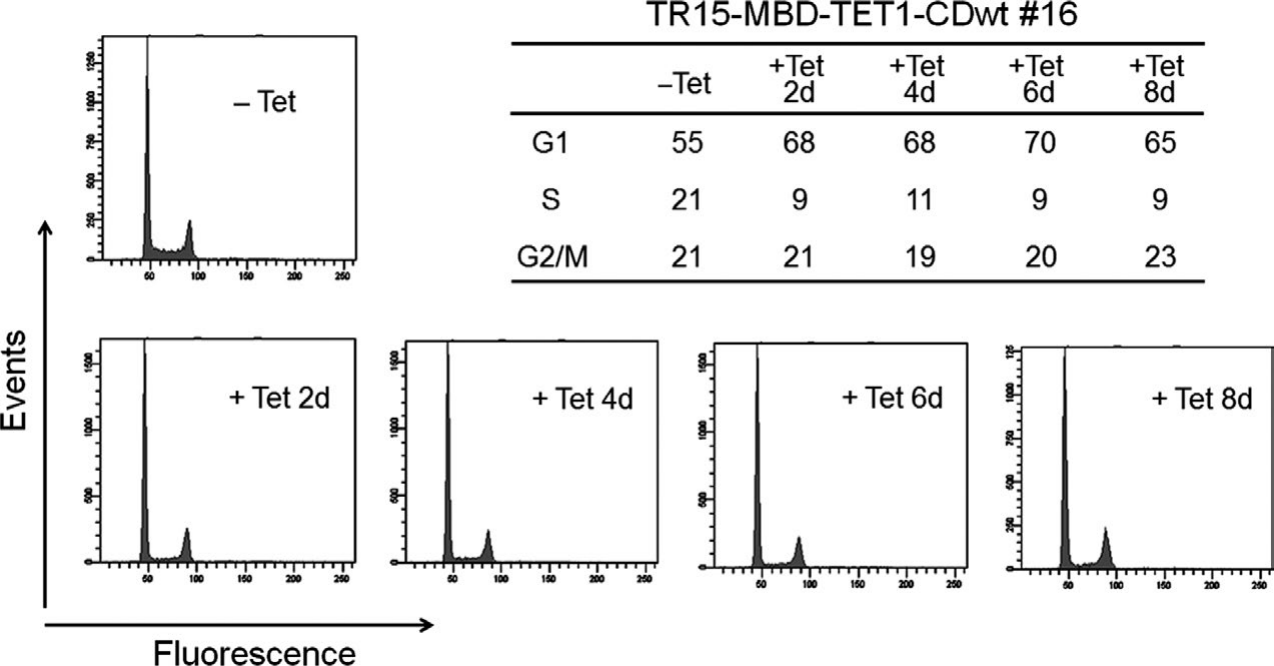
Inducible expression of MBD‐TET1‐CDwt decreases cells in S phase and increases ones in G1 phase in LNCaP cells. TR15‐MBD‐TET1‐CDwt #16 cells were treated with tetracycline (Tet) for the indicated time or without Tet. The cell cycle distribution was analyzed by flow cytometry.

Flow cytometry analysis of LNCaP_TR15‐MBD‐TET1‐CDwt #16 cells demonstrated that growth suppression is not caused by induction of apoptosis, but mainly by G1/S arrest (Fig. 6); again, the MBD‐TET1‐CDwt cell line LNCaP_TR9‐MBD‐TET1‐CDwt #17 showed basically the same results (Fig. S5).

## Discussion

DNA demethylation has been induced by DNMT inhibitors or disruption of the *DNMT* gene in a wide variety of cancer cells 28. These DNMT‐dependent methods often show phenotypic changes such as growth suppression and apoptosis in cancer cells. However, DNMTs have DNA methylation‐independent transcriptional repression activity 6, 7 thus, cellular effects caused by DNMT inhibition might be quite irrelevant to DNA demethylation. In addition, DNMT inhibitors impair DNA synthesis by forming azacytosine base‐DNMT adducts and may affect cellular phenotypes 5. For these reasons, we tried to isolate the effects of DNA demethylation alone in cancer cells by using a new DNMT‐independent method; the TET catalytic domain is targeted at hypermethylated promoters through MBD to oxidize 5‐mC. These oxidized methylcytosines were successively converted to cytosines. Our results using this DNMT‐independent method strongly suggest that the reactivation of hypermethylated genes by DNA demethylation suppresses the growth of LNCaP prostate cancer cells, largely consistent with previous results using DNMT inhibitor kazinol Q 29. MBD‐TET1‐CDwt induced growth suppression without apoptosis; kazinol Q induced apoptosis in LNCaP cells. The difference in cell phenotypes induced by these two methods may be explained by differences in the DNA demethylation level or by the presence of other factors, including methylation‐independent gene reactivation and inhibition of DNA synthesis.

It is notable that 33 of 51 (65%) genes upregulated by fivefold or more in MBD‐TET1‐CDwt expressing HEK293T cells were included in Aza‐CR‐mediated upregulated genes (see Fig. 3). Because both methods are based on DNA hypomethylation, the frequency of commonly upregulated genes tends to be higher. Using the MeTA method, on the other hand, the spectrum of selected genes differs, because this method upregulates hypermethylated genes without DNA demethylation; Aza‐CR upregulated genes classified as cancer/testis (CT) antigen such as MAGE (melanoma antigen), GAGE (G antigen), and XAGE (X antigen), whereas MeTA did not upregulate these genes 15. However, MBD‐TET1‐CDwt transfectants did upregulate these CT antigen genes. The majority of CT antigen genes contain short CGIs defined by Gardiner‐Garden and Frommer at their promoter regions. These results may suggest that effector molecules such as TET1‐CDwt and NFκB‐TAD can control the key that determines whether the hypermethylated genes are activated even though MBD always targets CGIs in the CT antigen genes. A future question remains of how the effectors differ in their activation of hypermethylated genes.

Recently, customizable transcription activator‐like effector (TALE) proteins and TET1 catalytic domain enabled targeted DNA demethylation; 30 this kind of technology can clarify the biological significance of individual hypermethylation‐mediated silenced genes. Our targeted DNA demethylation system has created another system for genome‐wide global demethylation using MBD, rather than TALE proteins. This newly developed system, MBD‐TET1‐CDwt, will discover yet to be identified methylated genes critical for cellular phenotypes in cancer. For instance, the common upregulated gene(s) in several prostate cancer cell lines by MBD‐TET1‐CD system will provide clues to clarify gene(s) associated with cellular proliferation. In addition, our global DNA demethylation system will help understanding of the role of aberrant CGI hypermethylation in cancer 31, 32, 33.

## Conflict of Interest

The authors have no conflict of interest to declare.

## Supporting information


**Figure S1.** Schematic diagram of five fusion constructs used in this study and catalytic activity of TET1‐CDwt.

**Figure S2.** An upward shift of gene expression in MBD‐TET1‐CDwt stable cells.

**Figure S3.** Bisulfite sequencing analyses of *MAGEA8* and *MAGEA9* genes in HEK293T and MBD‐TET1‐CDwt‐expressing HEK293T cells.

**Figure S4.** Cell proliferation assay of LNCaP_TR9‐derived cells.

**Figure S5.** Flow cytometry analysis of LNCaP_TR9‐MBD‐TET1‐CDwt #17 cells.

**Table S1.** Properties of genes upregulated by MBD‐TET1‐CDwt.

**Table S2.** Nucleotide sequences of primers used in bisulfite sequences.
