# Supplementary figures and images for: Targeted TET oxidase activity through methyl‐CpG‐binding domain extensively suppresses cancer cell proliferation
Source: Cancer Med. 2016 Jul 25;5(9):2522–33. doi: 10.1002/cam4.830 (PMC5055179; doi:10.1002/cam4.830)

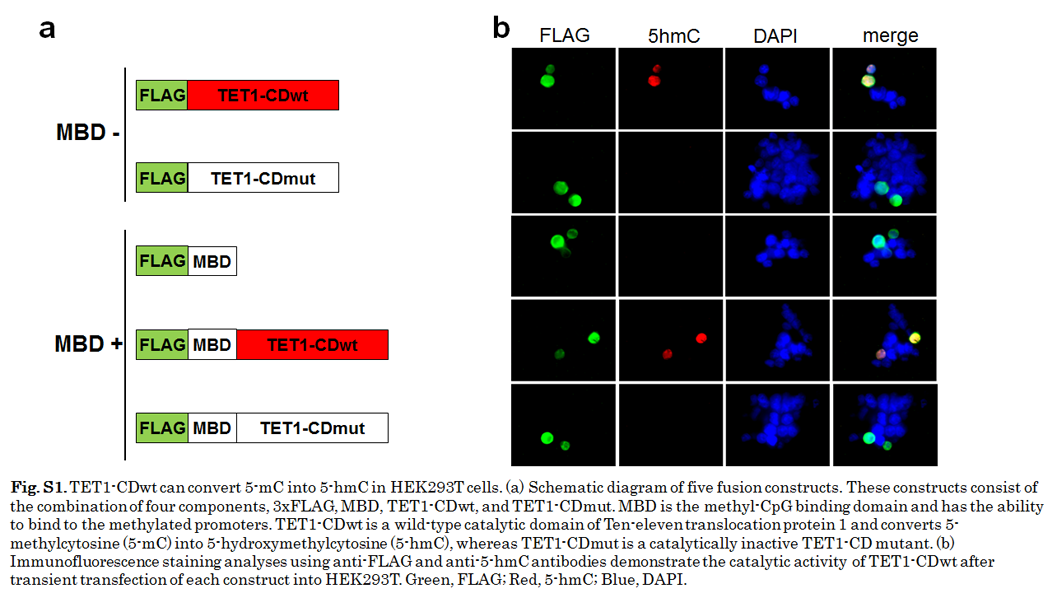

Supplement: Supplementary file 1 — Figure S1. Schematic diagram of five fusion constructs used in this study and catalytic activity of TET1‐CDwt. [file CAM4-5-2522-s001.tif]

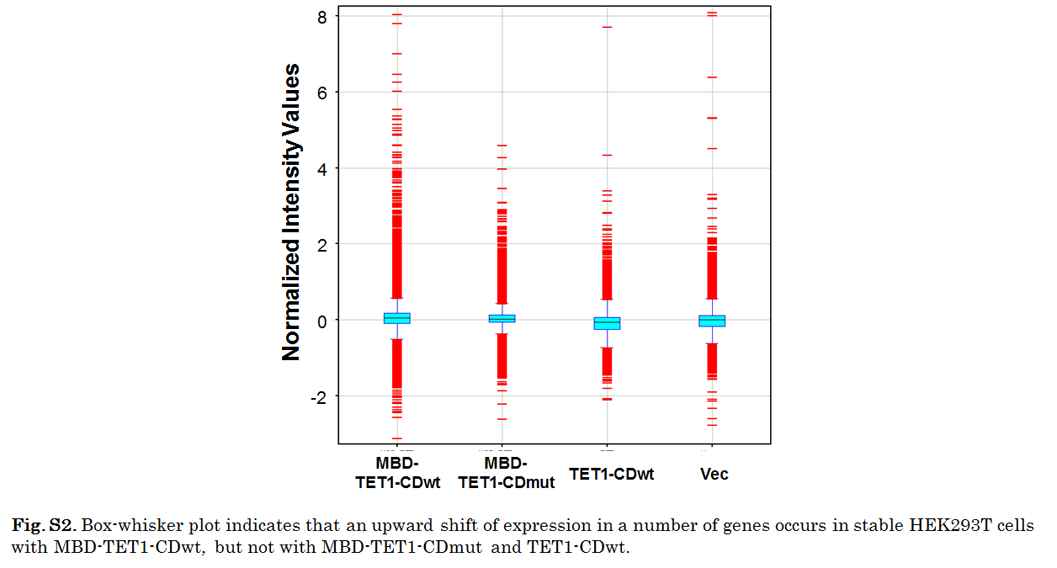

Supplement: Supplementary file 2 — Figure S2. An upward shift of gene expression in MBD‐TET1‐CDwt stable cells. [file CAM4-5-2522-s002.tif]

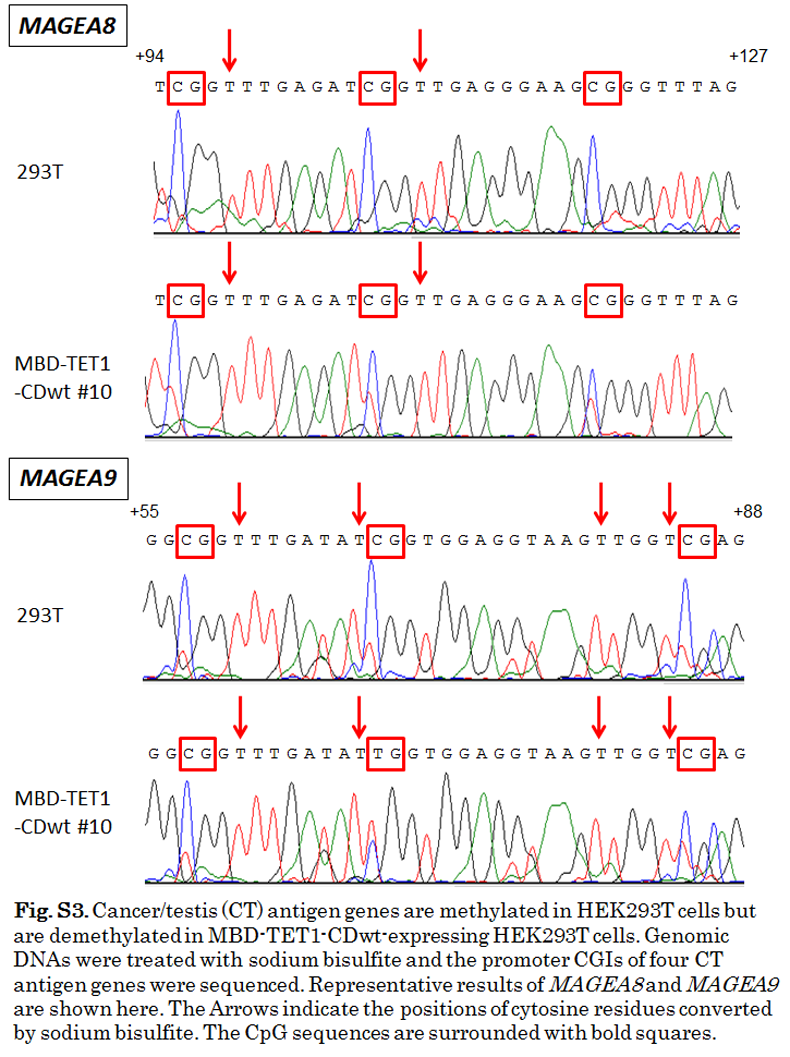

Supplement: Supplementary file 3 — Figure S3. Bisulfite sequencing analyses of MAGEA8 and MAGEA9 genes in HEK293T and MBD‐TET1‐CDwt‐expressing HEK293T cells. [file CAM4-5-2522-s003.tif]

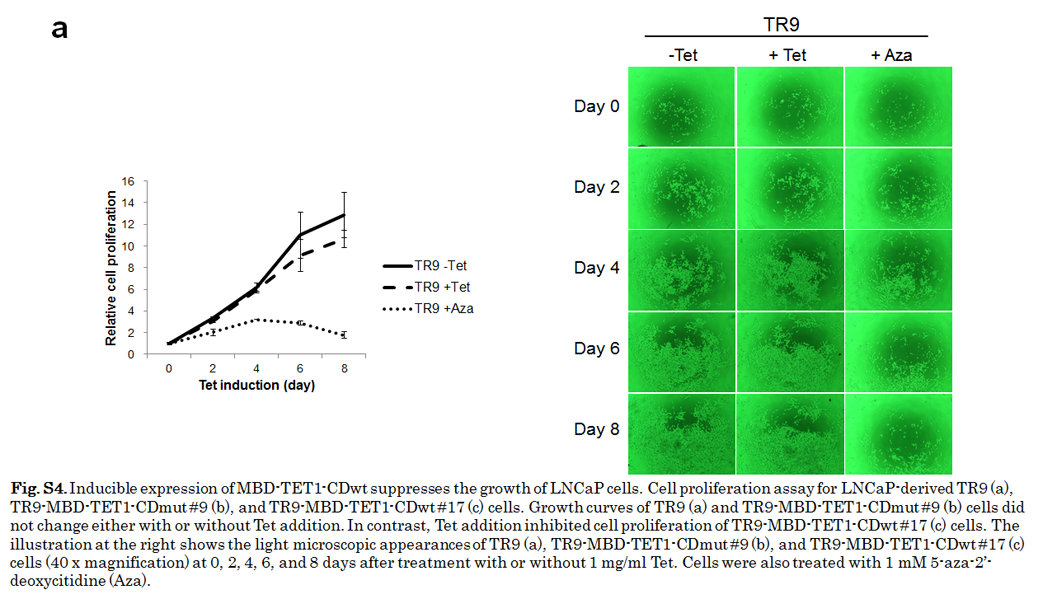

Supplement: Supplementary file 4 — Figure S4. Cell proliferation assay of LNCaP_TR9‐derived cells. [file CAM4-5-2522-s004.tif]

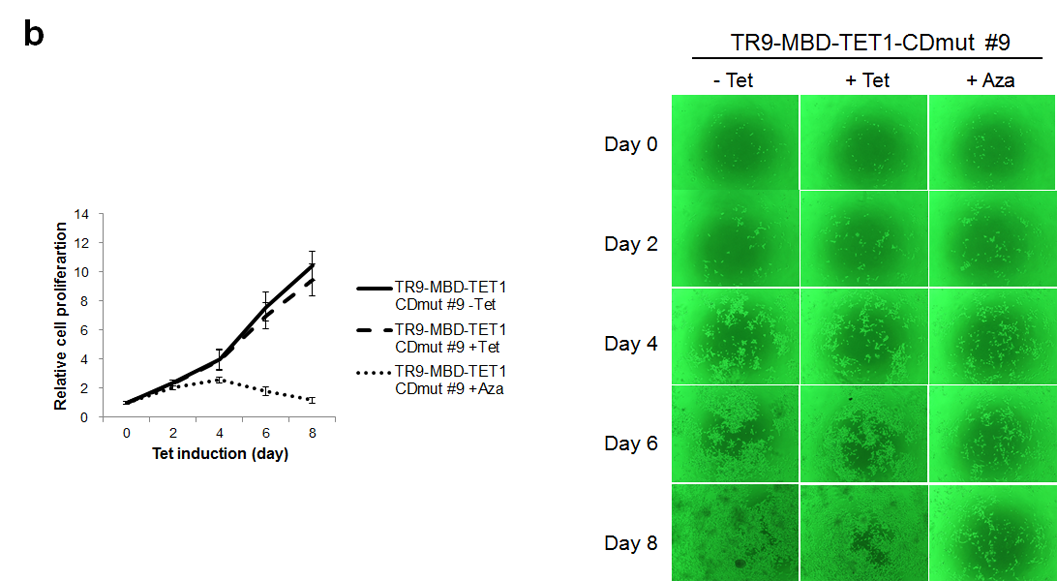

Supplement: Supplementary file 5 [file CAM4-5-2522-s005.tif]

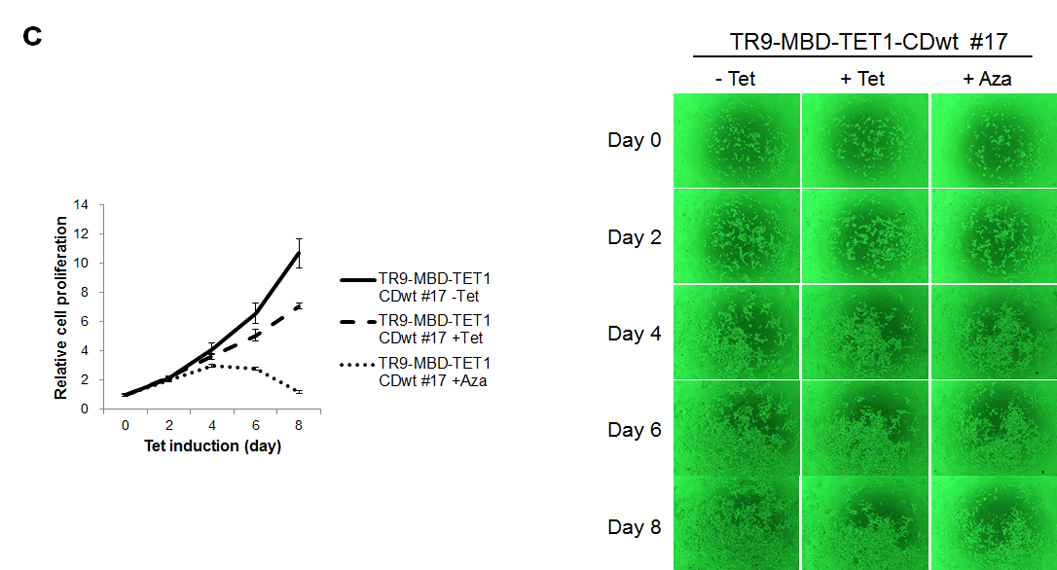

Supplement: Supplementary file 6 [file CAM4-5-2522-s006.tif]

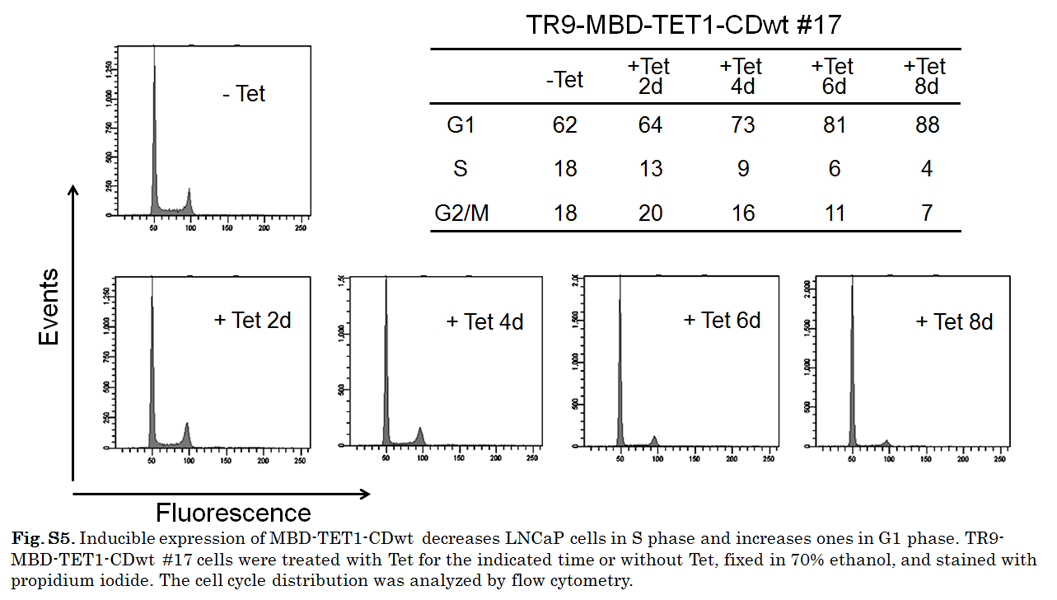

Supplement: Supplementary file 7 — Figure S5. Flow cytometry analysis of LNCaP_TR9‐MBD‐TET1‐CDwt #17 cells. [file CAM4-5-2522-s007.tif]
